# Supplementary material for: Residue-resolved monitoring of protein hyperpolarization at sub-second time resolution
Source: Commun Chem. 2021 Oct 22;4:147. doi: 10.1038/s42004-021-00587-y (PMC9814832; doi:10.1038/s42004-021-00587-y)
Supplement: Supplementary file 1 — Supplementary Material [file 42004_2021_587_MOESM1_ESM.pdf]

Residue-resolved monitoring of protein hyperpolarization at sub-second time resolution

Mattia Negroni[1], Dennis Kurzbach[1,*]

[1] University Vienna, Faculty of Chemistry, Institute of Biological Chemistry, Währinger Str. 38, 1090 Vienna, Austria
[*] Corresponding author: dennis.kurzbach@univie.ac.at

Supplementary Information

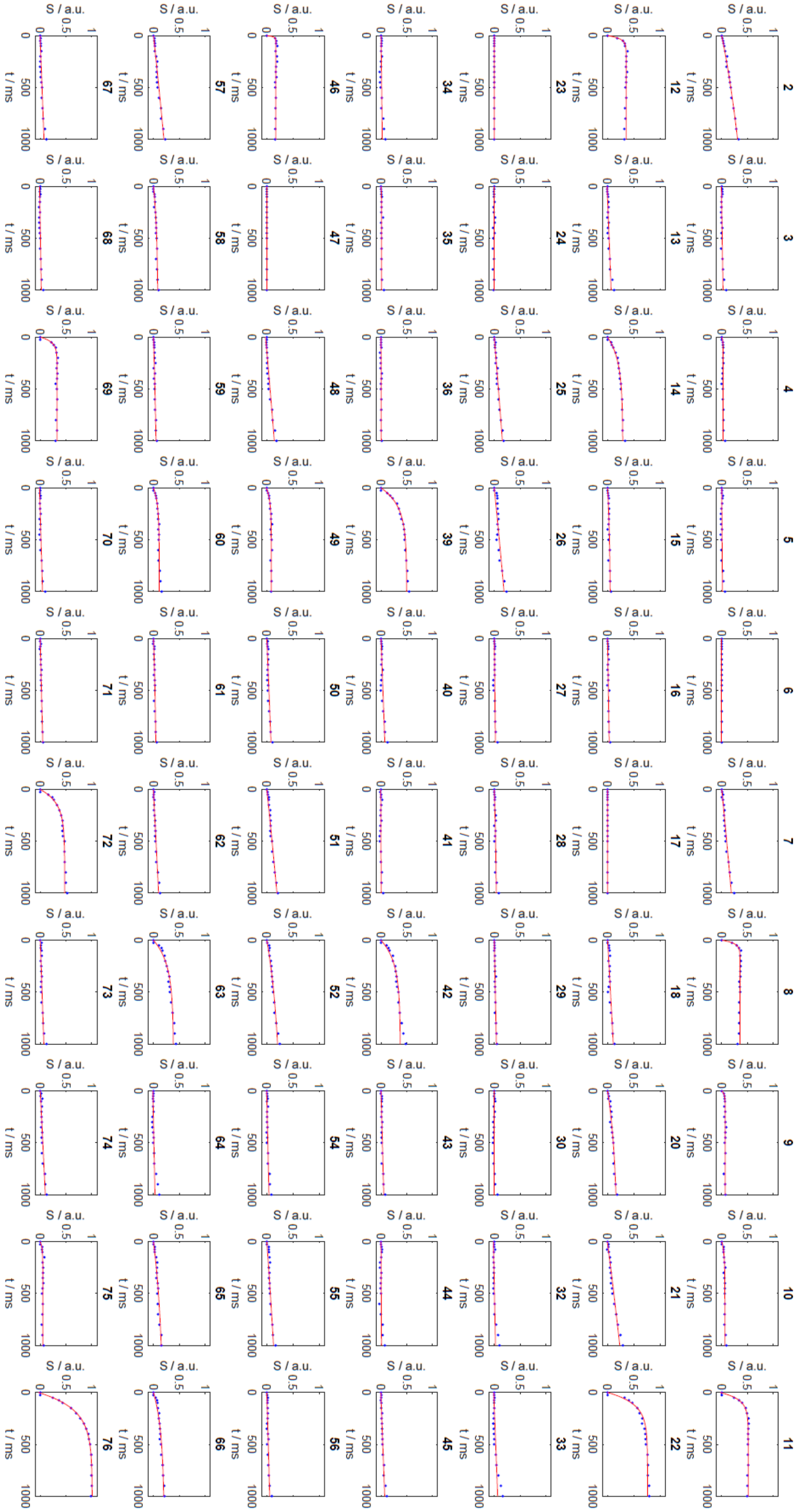

Supplementary Figure 1. Signal intensities for different mixing times in WS-NOESY experiments (Figure 2a of the main text shows the pulse sequence). The number above each panel indicates the residue index in Ubq. The time axis is logarithmic for better visibility.

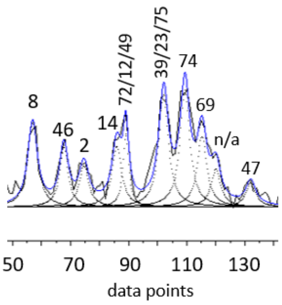

Supplementary Figure 2. Example for the deconvolution of the 10 Lorentzians. The spectrum was detected 4 seconds after mixing. The signal assignment is indicated. (cf. Figure 3a of the main text)

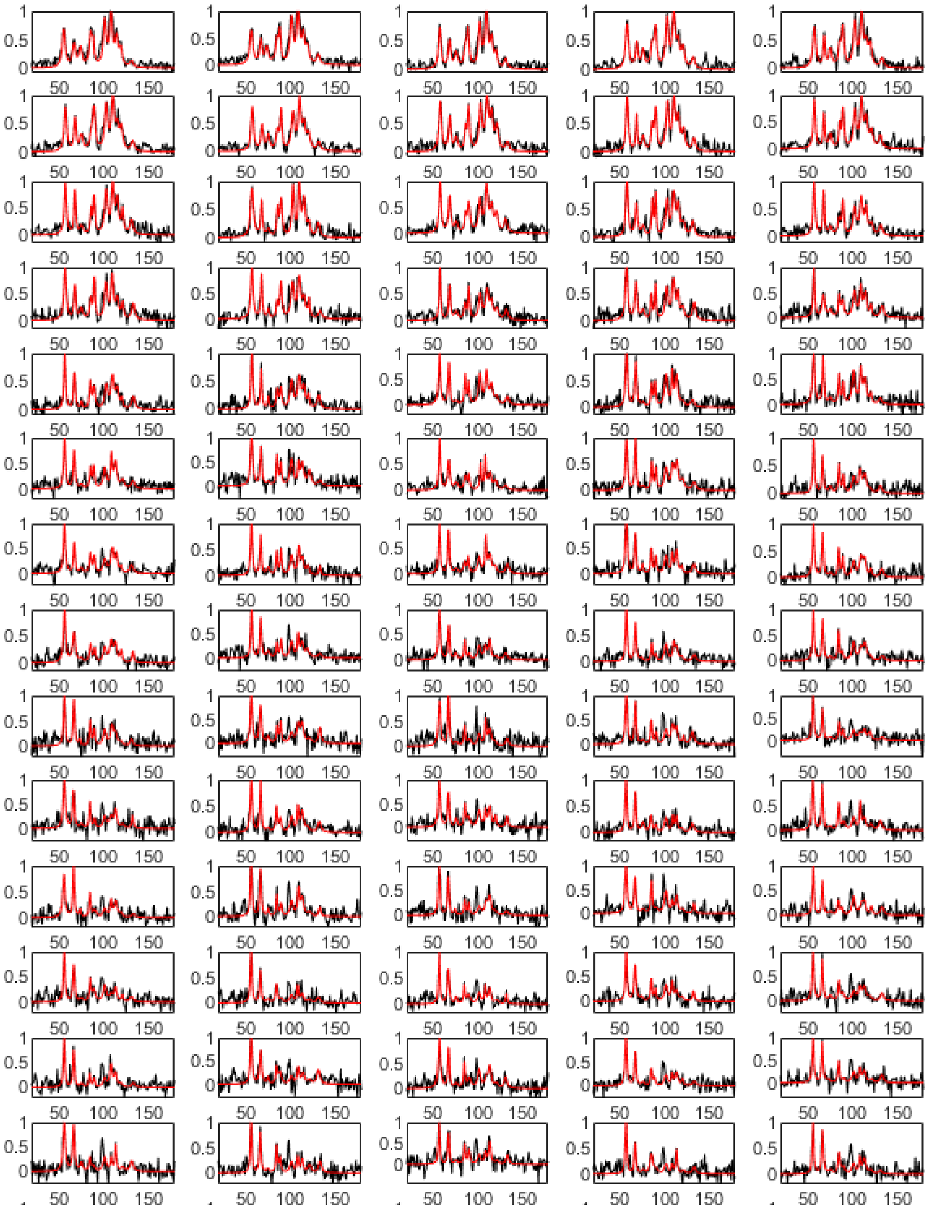

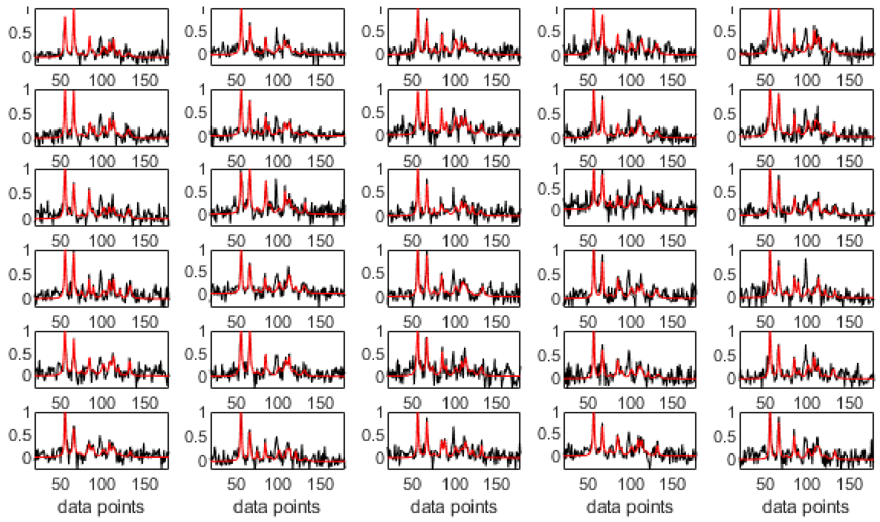

Supplementary Figure 3. Spectra (black) and fits to 10 Lorentzian lines (red) of the experimental *d*-DNP spectra. Note how most signals disappear towards the end of the detection period. The interscan delay was 0.5 s. The first spectrum has been detected 3 s after mixing.

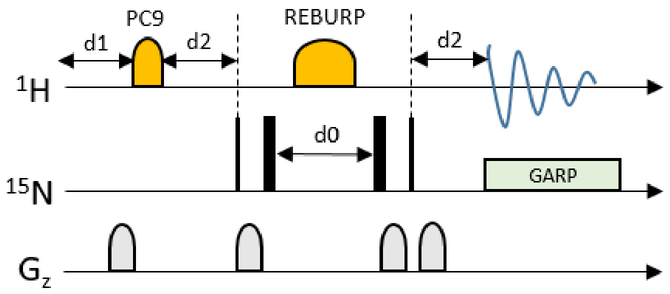

Supplementary Figure 4. Pulse sequence used for detecting the ^{15}N-edited ^{1}H-1D time-series of Ubq. The rectangular shapes indicated 90˚ pulses. For the ^{1}H channel a selective 1000 μs long PC90 pulse was used for 90° excitation and a 2000 μs long REBURP pulse was used for inversion. The carrier frequency was set to 10 ppm to avoid pulsing on the water resonance. Hence, only signals with chemical shifts >8 ppm were detected. d2 was set to 0.00345 s, d1 to 0.5 s and d0 to 0.00002780 s. d0 was not incremented. The FID was detected for 0.1 s during GARP decoupling.
